# Contributing to an autism biobank: Diverse perspectives from autistic participants, family members and researchers

**DOI:** 10.1177/13623613231203938

**Published:** 2023-10-26

**Authors:** Rozanna Lilley, Hannah Rapaport, Rebecca Poulsen, Michael Yudell, Elizabeth Pellicano

**Affiliations:** 1 Macquarie University, Australia; 2 Arizona State University, USA; 3 University College London, UK

**Keywords:** autism, biobanks, bioethics, biomarkers, genetics

## Abstract

There has been intense debate within the autistic and autism communities about the use of autism biobanks – repositories containing biological and phenotypic materials – and of genomic autism research more broadly. Here, we sought to understand the views and experiences of those contributing to one specific biobank, the Australian Autism Biobank. We adopted a multi-informant approach, conducting semi-structured interviews with 77 people, including 18 autistic probands, 46 parents and seven siblings, all of whom donated material to the Biobank, as well as six researchers employed on the project. Specifically, we asked: what motivated participants and researchers to contribute to the Australian Autism Biobank? And how did they feel about their involvement in that process? We analysed the data using reflexive thematic analysis, adopting an inductive approach within an essentialist framework. We identified three themes, which revealed a wide diversity of viewpoints, including positive conceptualisations of autism linked to neurodiversity and more negative conceptualisations linked to hopes for the development of genetic screening and reproductive choice. These findings have implications for the meaning, value and future directions of autism science. This research has been conducted using the Australian Autism Biobank resource.

**Lay abstract:**

A lot of autism research has focused on finding genes that might cause autism. To conduct these genetic studies, researchers have created ‘biobanks’ – collections of biological samples (such as blood, saliva, urine, stool and hair) and other health information (such as cognitive assessments and medical histories). Our study focused on the Australian Autism Biobank, which collected biological and health information from almost 1000 Australian autistic children and their families. We wanted to know what people thought about giving their information to the Biobank and why they chose to do so. We spoke to 71 people who gave to the Biobank, including 18 autistic adolescents and young adults, 46 of their parents and seven of their siblings. We also spoke to six researchers who worked on the Biobank project. We found that people were interested in giving their information to the Biobank so they could understand why some people were autistic. Some people felt knowing why could help them make choices about having children in the future. People also wanted to be involved in the Biobank because they believed it could be a resource that could help others in the future. They also trusted that scientists would keep their information safe and were keen to know how that information might be used in the future. Our findings show that people have lots of different views about autism biobanks. We suggest researchers should listen to these different views as they develop their work.

Efforts to identify the biological underpinnings of autism generate significant controversy within the autistic and autism communities. Such attempts to advance understanding of autism – both syndromic and idiopathic (Abrahams & Geschwind, 2008; Buxbaum, 2022; Richards et al., 2015) – are internationally supported by repositories of biological material, and other personal information known as biobanks (Organisation for Economic Cooperation and Development, 2007). Among conventional scientists, these biobanks are routinely described as invaluable assets (Kinkorová, 2016). Yet, autism-specific biobanks, including the US-based Autism Genetics Resource Exchange, the Autism Tissue Program, the Simons Simplex Collection and the Spectrum 10K project, each of which claim to contribute towards improving knowledge of autism causation as well as informing diagnostic and treatment discoveries (Alvares et al., 2018; Reilly et al., 2017), have attracted extensive criticism from some autistic and neurodiversity advocates (Chapman et al., 2021).

There are complex historical and contemporary reasons for this controversy. In particular, the identification of genes related to autism has fuelled concern that such knowledge could be used for embryo selection or pregnancy termination. The search to prevent or cure autism, once an explicit aim of some autism research, is now considered by many in the research and advocacy communities to be undesirable, and some advocacy organisations have sharply distanced themselves from it (Pellicano et al., 2011; Singh, 2015a). Fears may also be fostered by the expansion of prenatal testing in general leading to concerns that autism could be the next prenatal genetic test (Rothschild, 2005; Thomas et al., 2021). Such concerns have also fuelled the ‘expressivist objection’ (Buchanan, 1996) that genetic research can devalue existing disabled and neurodivergent lives (Boardman, 2014, 2021; see Snyder & Mitchell, 2006).

Despite these controversies, existing studies reveal that many parents of autistic children participate in genetic research because they believe it can help explain the causes of autism, assist reproductive decision-making and improve diagnosis and treatments (Asbury et al., 2023; Chen et al., 2013, 2015; Reiff et al., 2017; Tabor et al., 2011; Xu et al., 2016, 2018 see also Hens et al., 2016b; Trottier et al., 2013). Further studies suggest that many researchers and parents of autistic children seek disclosure of genetic results (Fischbach et al., 2016; Johannessen et al., 2016, 2017; Li et al., 2022; Miller et al., 2010; Zhang et al., 2022) – even given the limited predictive power of current genetic testing (Miller et al., 2010). In the most detailed sociological account of the views of parents of autistic children donating to an autism biobank, Singh (2015a, 2015b) found a range of important motivators, including a sense of obligation and the need for diagnostic evaluation to access services. Furthermore, she found parents ‘placed a high level of trust’ in clinical research entities collecting biobank data (Singh, 2015a, p. 141). This strong faith in conventional science appears to persist despite uncertainties about the uses to which genetic information might be put (Bumiller, 2009; Byres et al., 2023).

Alongside these perceived benefits, studies with parents of autistic children have also revealed concerns, including the potential for genetic discrimination by employers and insurance companies, and worries about data security (Biesecker et al., 2021; Johannessen et al., 2017; Madrid et al., 2022; Wagner et al., 2020; Yudell et al., 2013), the stress of blood draws for children (Hens et al., 2016a) and the limited practical benefit of genetic studies (Chen et al., 2013). Some research also reports parental concerns that genetic information used for family planning may harm autistic children by contributing to stigma (Byres et al., 2023; Tabor et al., 2011).

## This study

While these studies have helped our understanding of people’s experiences, research remains limited. Importantly, there has been little research involving autistic participants, who themselves have contributed biological material. Our aim, therefore, was to investigate these issues in-depth, focusing on participants’ contribution to one biobank, the Australian Autism Biobank, run by the Cooperative Research Centre for Living with Autism (Autism CRC). The Australian Autism Biobank collected biospecimens (blood, stool, hair, urine and sometimes saliva) from autistic children and their families and phenotypic data (clinical assessments and questionnaires) from 2013 to 2018 (Alvares et al., 2018). In total, 929 autistic children participated as well as 239 probands from an existing Western Australian autism biobank, following reconsent. Parents, siblings and non-autistic children also provided data (Eapen & Masi, 2020). Phenotypic data were stored in the Australian Autism Biobank database on a central server; biological data were processed and placed in long-term biobanking storage facilities. Access to these data is subject both to a data access fee, and approval by a Data Access Committee, which includes representatives from the Autism CRC, researchers and the autistic community (see Alvares et al., 2018, for overall study protocol).

To examine the motivations and experiences of those contributing to this Biobank, we adopted a multi-informant approach, conducting semi-structured interviews with autistic probands, parents, siblings and researchers. We asked: what motivated participants and researchers to contribute to the Australian Autism Biobank? And how did they feel about that process? We did so with the expectation that analysing the narratives of Biobank participation would offer a unique contribution to understanding attitudes towards autism science and the ‘contextual bioethics’ (Singh, 2015b) of participants and researchers at a time of intense debate.

## Methods

### Participants

Seventy-seven people participated. To recruit families, Biobank staff identified those who had (1) participated in the Biobank and (2) provided consent to participate in future research (*n* = 643). The Biobank lead investigator emailed families inviting them to contact researchers if they would like to participate. To recruit researchers, we contacted all site leads for the Biobank and recruitment emails were sent to researchers employed on the project. Six agreed.

Of the consenting families, we interviewed 71 family members, including 46 parents/carers, 18 autistic children and adults and 7 siblings.

#### Parents

Of the 46 parents/primary carers (hereafter ‘parents’), we interviewed 40 mothers, 3 fathers and 3 adoptive parents (grandparents and a mother). Most were women (*n* = 42; 90%) of white ethnic background and moderate-to-high socioeconomic status (see Table 1). A significant minority reported mental health conditions, especially depression (*n* = 12; 26%) and anxiety (*n* = 10; 22%). Five mothers and one father (13% of the parent sample) self-identified as autistic.

**Table 1.** Parents’ background characteristics.

	Mean (SD) Range or *N* (%)
Mothers’ age (in years)^a^	43.98 (6.33) 30.65–57.39
Mothers’ predominant ethnic background	
Aboriginal	1 (2%)
Asian	1 (2%)
Caucasian	40 (87%)
Mixed (Caucasian and Jamaican)	1 (2%)
Missing	3 (6%)
Location in Australia	
New South Wales	7 (15%)
Queensland	10 (22%)
Victoria	12 (26%)
Western Australia	17 (37%)
Highest qualification	
Completed less than 10 years of school	1 (2%)
Completed 10 years of school	1 (2%)
Completed 12 years of school	3 (6%)
Completed trade or technical qualification	10 (22%)
University degree	28 (41%)
Missing	3 (6%)
Total annual family income (before tax) in $AUD	
35,001 to 40,000 (674–769 per week)	1 (2%)
40,001 to 50,000 (770–962 per week)	2 (4%)
50,001 to 60,000 (963–1154 per week)	1 (2%)
60,001 to 70,000 (1155–1346 per week)	3 (6%)
70,001 to 78,000 (1347–1500 per week)	1 (2%)
78,001 to 104,000 (1501–2000 per week)	4 (9%)
104,001 or more (> 2001 per week)	28 (61%)
Prefer not to say	1 (2%)
Missing	5 (11%)
Number of siblings with autism diagnosis	22
Relatives with developmental difficulties	
Yes	29 (63%)
No	17 (37%)
Co-occurring conditions^b^	
Anxiety disorder	10 (22%)
Asthma	7 (15%)
Chronic allergies	5 (11%)
Depression	12 (26%)
Diabetes	4 (9%)
Epilepsy	2 (4%)
Heart disease	1 (2%)
Hepatitis	2 (4%)
Heart disease	1 (2%)
Hypertension	5 (11%)
Polycystic ovary syndrome	5 (11%)
Thyroid problems	4 (9%)

a *n* = 4 missing.

b Percentages may not sum to 100% due to rounding issues.

Parents reported on 46 autistic ‘probands’, including 39 males (85%) and 7 females (15%). Most children were given a formal diagnosis of an autism spectrum condition during the early years (M age = 4.69, SD = 2.21). Most (*n* = 36; 74%) had also received additional diagnoses, including one-third with co-occurring intellectual disability (ID) or global developmental delay (*n* = 16; 35%). Children’s scores on the Vineland Adaptive Behaviour Scale (Sparrow et al., 2005) and the Autism Diagnostic Observation Scale (ADOS; Lord et al., 1999, 2012) at the time of Biobank participation confirmed substantial variation in adaptive functioning and autistic features (see Table 2).

**Table 2.** Characteristics of the autistic probands (*n* = 46), and of participating autistic young people (*n* = 18), as reported by parents.

	Autistic probands (*n* = 46)	Participating autistic young people (*n* = 18)
	Mean (SD) Range or *N* (%)	Mean (SD) Range or *N* (%)
Nature of relationship to proband		
Biological parent	44 (96%)	16 (89%)
Biological grandparent	1 (2%)	1 (5%)
Foster parent	1 (2%)	1 (5%)
Proband child’s age (in years)	8.38 (4.08) 3.00–17.81	15.90 (3.07) 12–22
Proband child’s sex		
Female	7 (15%)	2 (11%)
Male	39 (85%)	16 (89%)
Autism diagnosis		
Autism	41 (89%)	14 (78%)
PDD-NOS	2 (4%)	2 (11%)
Asperger’s	3 (6%)	2 (11%)
Age of autism diagnosis (in years)	4.69 (2.21) 2–11	6.0 (2.31) 3–11
Co-occurring diagnoses		
Intellectual disability	5 (11%)	2 (11%)
Global developmental delay	11 (24%)	3 (17%)
Epilepsy	3 (6%)	1 (5%)
Otitis Media	8 (17%)	4 (22%)
Other	13 (28%)^a^	6 (33%)^b^
None of the above	12 (26%)	6 (33%)
Vineland Adaptive Behaviour Composite^c^	75.92 (14.87) 44–118	73.75 (10.94) 54–90
Vineland adaptive level		
Low	14 (30%)	6 (33%)
Moderately low	19 (41%)	9 (50%)
Adequate	6 (13%)	1 (5%)
Missing	7 (15%)	2 (11%)
ADOS-G or ADOS-2 Module^d^		
Module 1	8 (17%)	0
Module 2	7 (15%)	2 (11%)
Module 3	25 (54%)	12 (67%)
Module 4	4 (9%)	4 (22%)
ADOS Calibrated Severity Score (score out of 10)^e^	6.68 (1.72) 2–10	6.44 (1.79) 2–9

PDD-NOS: pervasive developmental disorder – not otherwise specified; ADOS-G: Autism Diagnostic Observation Schedules – Generic; ADOS-2: Autism Diagnostic Observation Schedules – 2nd edition.

a Other diagnoses included anxiety disorder, sleep disorder, attention deficit hyperactivity disorder (ADHD), depression, obsessive compulsive disorder (OCD) and dyspraxia.

b Other diagnoses include anxiety disorder, ADHD, OCD, depression and dyspraxia.

c Vineland Adaptive Behaviour Scale (Sparrow et al., 2005).

d ADOS-G (Lord et al., 1999), ADOS-2 (Lord et al., 2012).

e *n* = 2 missing.

#### Autistic children and adults

We interviewed 18 of the 46 autistic probands (39%; 2 females, 16 males), including 14 children and 4 young adults, ranging from 12 to 22 years. All had received an independent clinical diagnosis of an autism spectrum condition, on average, at 6 years. Most young people (*n* = 12; 66%) had co-occurring neuropsychiatric/developmental conditions (see Table 2).

#### Siblings

We interviewed five sisters and two brothers from 5 families, ranging from 12 to 18 years. None had received an autism diagnosis at the time of Biobank participation, although one (14%) reported a subsequent diagnosis of autism and two (28%) of attention deficit hyperactivity disorder (ADHD).

#### Researchers

Six researchers participated from three different Biobank sites. No demographic information is reported to protect participants’ anonymity.

### Procedure

Ethical approval for the study was obtained from Macquarie University’s Human Research Ethics Committee (Ref. no: 5201832864200). All participants, including young people, provided written informed consent before taking part; parents provided additional consent for their children.

#### Background information

To reduce the potential burden from over-testing and avoid duplication of assessments, we sought permission to access families’ background data from the Biobank database, including demographics, family history, proband autistic features, as measured by the ADOS (Lord et al., 1999, 2012) and adaptive functioning, as measured by the VABS (Sparrow et al., 2005; see Tables 1 and 2). Biobank staff provided de-identified data in October 2020. We note these data represent retrospective, rather than concurrent, information on participants.

#### Interviews

Participants took part in semi-structured interviews between November 2019 and February 2020. Most were face-to-face (*n* = 65; 90%), with the remainder conducted by telephone (*n* = 7; 10%). Interviewees were asked open-ended questions about their experiences of Biobank participation; their perceptions on the causes of autism; their understanding of, and attitudes towards, genetic research; and their views of autism research more broadly. The questions were reworded to suit their different roles (see Supplementary Materials for interview schedules). Prompt questions were used to elicit further information. We provided participants with the primary interview questions ahead of the interview. Young people had the option of being interviewed alongside their parent (*n* = 12; 67%).

There were 41 h 17 min of interview material (range = 6–83 min per interview). All interviews were recorded and transcribed verbatim, except for one (researcher) interviewee, who preferred interviewer note-taking to recording. Participants’ transcripts were returned to them for review to check for accuracy and remove any potentially identifying details. All non-researchers were given gift vouchers to thank them for their time.

### Data analysis

We followed Braun and Clarke’s (2019) method for reflexive thematic analysis within an essentialist framework, in which our goal was to report the meanings and experienced reality of the participants. We adopted an inductive (bottom-up) approach (i.e. without integrating the themes within any pre-existing coding schemes or preconceptions) to identify patterned meanings within the data set. Our analytic approach was informed by our training in anthropology (RL), education (EP), psychology (EP, HR), neuroscience (RP) and public health (MY); positionalities as an autistic researcher (RP) and parent of an autistic child/adult (RL, RP); and a neurodiversity lens to autism research and practice (Chapman & Botha, 2022; Pellicano & den Houting, 2022).

Our analysis began during the interview process, as RL took reflexive notes immediately following each interview, including a content summary as well as striking observations and reflections. RL read and re-read the transcripts, discussing potential codes with EP and with the broader team fortnightly, before applying codes to all transcripts. Data from all participants informed the final coding framework. RL then generated a draft thematic map, before sending it to the team for discussion. The team liaised several times to review the themes and subthemes, focusing on semantic features of the data (staying close to participants’ language), resolving discrepancies, and deciding on the final descriptions. Analysis was therefore iterative and reflexive (Braun & Clarke, 2019).

### Community involvement

The lead author is the mother of an autistic adult. This experiential expertise informed the design, conduct and analysis of the interviews. At the analysis phase, it became clear that autistic experiential expertise would also be beneficial. We therefore invited RP, an autistic neuroscientist, to join. During analysis, the team, including RP, met fortnightly to analyse the data, during which we read and reflected upon each transcript and discussed potential codes and themes. Team members were also encouraged to communicate their views and reflections over email and on corresponding documents. The experiential expertise of both RP and RL was integral to the study.

## Results

**Figure 1.**
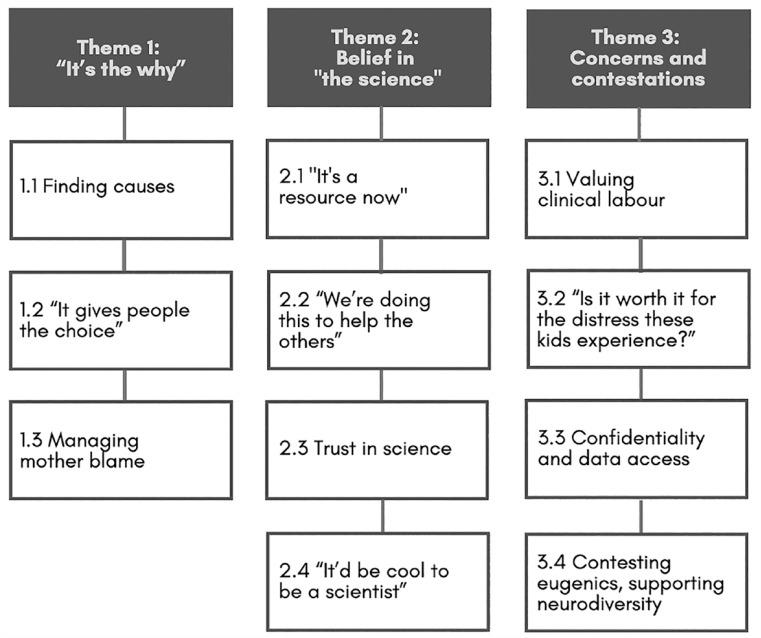
Themes and subthemes identified in our interviewees’ reflections on Biobank participation.

We identified three themes (see Figure 1). We use the terms ‘participants’ to refer to those who contributed biological and phenotypic data to the Biobank (P = parents; A = Autistic adolescents/adults); ‘researchers’ (R) to either employees responsible for collecting data or site leads; and ‘interviewees’, to refer collectively to participants and researchers. Readers are advised that some may find this material distressing, as it includes discussion of reproductive choice, eugenic implications and internalised stigma.

### Theme 1: ‘It’s the Why’

#### Finding causes

Parents often described their Biobank participation as motivated by a search for answers about autism causation. As one mother, parenting two autistic children with intellectual disability (ID), said: ‘It’s the why. Why does this happen? If your child’s got Down syndrome, you know they’ve had this . . . I think when you’re a parent of a child with autism you really have no idea why this has happened’ (P001). Another mother, when asked why she and her adolescent autistic son, attending a special school, decided to take part, replied: ‘We want answers . . . We want to know why this is happening’ (P002).

Most interviewees were focused on genetic causation. One autistic adolescent said the purpose of the Biobank is to discover ‘maybe what causes autism and if it can be passed down’ (A004). A researcher commented: ‘There’s some sort of genetic inherited something-or-other there . . . I’d love to figure it out in this lifetime’ (R005). Some linked the importance of ‘understanding the causative factors’ (P026) to the increased prevalence of neurodevelopmental conditions: ‘I’m hoping they find a reason for it. Because it’s so prevalent now, and so many people I know whose children have autism or ADHD’ (P024).

For others, the Biobank’s presumed focus on causation included a range of potential factors (‘genetics, environmental . . . ’.; P036). In general, participants thought that the Biobank would contribute to research that will ‘crack the code eventually’ (P042).

#### ‘It gives people the choice’

Participants believed that research using biobank data will eventually result in the identification of autism-specific biomarkers, perceived as having multiple benefits. Some parents hoped for improved diagnostic outcomes, including ‘being able to diagnose people earlier’ (P019) and improved identification of autistic girls – ‘I think it’s harder to diagnose girls with autism’ (P011). Earlier identification was linked with the possibility of ‘early intervention support’ for children (R033). This same researcher suggested that the development of ‘preventive measures’ based on genetic screening might not ‘actually [be] that far away’. One parent agreed: ‘I think in the future it’ll be inevitable that you’ll be screened’ (P018).

Parents said they would have appreciated earlier identification to allow for better planning: ‘A heel prick test at birth, okay, you’re going to need to implement this, we’ll help you with this’ (P015). A researcher talked about the potential of biobank research to modify aspects of autism: ‘our genes are our genes, we can’t change them, but it’s that other modification stuff that we can do, and it can be non-pharmacological and pharmacological’ (R006).

The aim of identifying autism in pregnancy was described by some participants as part of reproductive decision-making: ‘To me, it’s just about choice . . . It’s like testing for Down syndrome’ (P001). The same mother added, ‘if we could have had a genetic test prior to having [child], we would not have continued’. Some parents suggested having this choice could ‘save someone a whole lot of grief’ (P016). Some mothers highlighted how challenging parenting their autistic children was, especially if they had co-occurring ID, suggesting they may remain forever ‘childlike’ (P012). Others were less certain about prenatal testing: ‘I don’t know, probably not, because you don’t know how severe they’ll be’ (P002). Parents also hoped that biobank research would assist their own autistic children to make reproductive choices (‘to maybe have kids or not have kids’; P002), as well as their siblings: ‘So maybe for my two older boys, if they understand why their brother has autism and they don’t’ (P024).

Autistic participants also raised the issues of prevention and cure. One autistic adolescent suggested that research might ‘potentially maybe prevent it [autism] in the future, or the more serious cases at least’ (A032). Another explained that the research was ‘trying to reduce the probability of autism occurring again in birth or genetic combination’ (A042). When asked whether he thought that was a good idea, he replied ‘To be honest, I do think it is’.

#### Managing mother blame

Mothers suggested that finding a cause for their child’s autism was linked to overturning stigma and alleviating guilt or self-blame. Some criticised psychodynamic theories of autism causation: ‘They used to say refrigerator mothers and all that. I think that’s rubbish’ (P020).

A sense of maternal culpability was linked to multiple competing theories of autism causation: ‘I’ve looked high and low about what could it have been. Was it that I had some antibiotics when I was pregnant? Was it because I had low iron? . . . Is it genetic? Is it something that’s in the family?’ (P039). Another mother, who reported being told that her age and history of drug-taking could be causative factors, commented: ‘If it was able to dispel some of the ignorant comments that I’ve been on the receiving end of, it would be a good thing’ (P041). Others, however, reported that psychogenic theories continued to impact maternal self-esteem. One mother from a multiplex family complained that her relatives blame her ‘for my gene pool’. She continues to wonder whether her mothering was partly responsible for her children being autistic: ‘So I blame myself for part of it and people go, ‘Oh autism’s genetic, why would you think that?’’ (P017).

### Theme 2: Belief in ‘the science’

Both participants and researchers spoke about their contributions to the Biobank as part of their broader commitment to ‘the science’ (P009).

#### ‘It’s a resource now’

While some participants saw the search for biomarkers as the main purpose of the Biobank, others stressed a broader range of research aims. One mother described how biobank collection could streamline research: ‘they just want them so that whenever they do research, they don’t have to go around doing blood and wee and all that’ (P020). The purpose of the Biobank as ‘pooled information’ (R006) for diverse scientific purposes was widely emphasised. One researcher said: ‘It’s a resource now’ (R029).

The emphasis on open aims was integral. One parent commented that the collected samples and data ‘will unlock a door for them in the future’ (P004). A researcher explained: ‘You take these biological specimens with the idea of future-proofing’ (R029).

#### ‘We’re doing this to help the others’

Participants described their motivations for being part of the biobank as primarily stemming from a desire to contribute to science and, in so doing, to help others: ‘I like to give back to science and I’m always interested in helping them do autism research’ (P020). A grandmother gave a strong sense of the intensity of that commitment when she spoke with her autistic grandson about the importance of contributing to research:

I said, ‘it won’t help us, but it will help the ones that come after . . . the reason you have got the level of assistance so far is from all the people that came before you that helped with research’. (P009)

Researchers explicitly acknowledged the dedication of participants: ‘They’re obviously going through many different challenges in their own lives, but are still willing to contribute . . . It’s very humbling’ (R033).

#### Trust in science

Some participants stressed they trust the scientific process – ‘You can prove and disprove things and science can do that’ (P001) – and in scientists’ expertise: ‘It’s like donating to St Vincent de Paul. I’ll just give you the money. You can just go and disperse it to whatever you want to do with it’ (P043).

Others expressed confidence in their own scientific expertise: ‘I’m a scientist so I highly believe in research’ (P011); ‘I was quite willing, just because of my medical background . . . you have to have scientific evidence to move forward’ (P022). The desire to contribute to scientific research was sometimes related to concerns about the credibility of some autism causation theories: ‘I think a lot of time and money is wasted on all these freaking conspiracy theories, like vaccinations and fluoride in the water’ (P011).

#### ‘It’d be cool to be a scientist’

Some parents stated that their autistic children enjoyed science and contributed to the Biobank so that they could learn more about how scientists conduct research. One mother commented that her son, who has ambitions to be either a doctor or a scientist, ‘loves that he participated, because he’s like, ‘otherwise if people don’t do research, Mum, people don’t learn’’ (P017). Another mother said that her son ‘was blown away’ when he went to a university to participate: ‘He’s just like ‘breathtaking – this is where I’m going’’ (P015).

Autistic adolescents also directly spoke about their interest in science and their enjoyment of participating in research. One 13-year-old even said: ‘Every day . . ., I always hoped that Mum would go, ‘Hey, there’s another test going on. Would you like to join in?’ That was my dream’ (A032). When asked why he enjoyed research participation so much, he replied ‘I just love knowledge’. Another adolescent related his interest in genetics to his ‘love for Jurassic Park . . . how they use genetics to de-extinct dinosaurs’ (A043). A 14-year-old discussed his career aspirations: ‘I’m trying to see if I want to become a scientist’ (A042).

### Theme 3: concerns and contestations

Despite most interviewees reporting strong support for the Biobank, they also raised concerns about potential ethical challenges.

#### Valuing clinical labour

Participants spoke about the importance of access to research results. Some families reported that access to summaries of phenotypic assessments motivated their participation: ‘The bit that 100 per cent sold me’ was the prospect of ‘an updated IQ test’ (P020). Parents described being offered research results: ‘Immediately after our diagnosis, we were approached to join the study. And the main sort of encouragement was that they would do a cognitive assessment and some extra blood work’ (P022). Parents who received results used these for school assessments (‘they’ve always been able to use that to shore up the case for funding’; P018) and insurance purposes. Other families, however, said they received no results.

Researchers mentioned that participation ‘was an opportunity to also get additional pathology requests done’ (R033). While the return of additional bloodwork results was an uncontroversial incentive, at some sites, researchers said returning behavioural results (such as the ADOS or VABS) was debated because not all people employed to collect phenotypic data were trained psychologists, and families needed to understand that summaries are not equivalent to professional clinical assessments. There was also concern that offering phenotypic assessment summaries as a direct incentive may be ethically complex or ‘a bit backwards’ (R005).

Importantly, participants also emphasised they wanted their research contributions to be respected and valued: ‘not just take our stuff and then piss off’ (P012). Some parents underlined the value of their own and others’ experiential expertise: ‘I think by participating, we’re giving our real-life experience’ (P038); ‘As a parent, I can go and tell people straight from the horse’s mouth’ (P041).

Researchers were sensitive to the importance of valuing the contributions of families. One cautioned against being ‘too transactional’, expanding on how ‘the notion of taking their precious biological specimens and then sending it off and not even seeing families just didn’t gel with me’ (R029). Some researchers believed that investing time in face-to-face meetings was one way of acknowledging the value of participants as people (rather than an ID number) and providing ‘the payoff of seeing the families’ (R029).

##### ‘Is it worth it for the distress these kids experience?’

One particular aspect of biobank participation – the donation of blood – was distressing for some participants and their families: ‘I had to hold him down screaming’ (P022); ‘The bloods is the most traumatic thing I’ve ever done . . . It was just the worst experience ever’ (P015). Indeed, one father described withdrawing from the study once he realised it involved a blood draw because he did not want his autistic daughter to be ‘pinned down’ (P030). While some autistic participants reported that the blood draw was not too upsetting – ‘I wasn’t too freaked out’ (A028) – others said they were ‘scared’ (A008).

Researchers detailed their efforts to provide a positive experience of blood draws including ‘doing all the distraction possible’ (R005). Nevertheless, some still expressed doubts about the ethics of taking blood from children: ‘Is it worth it for the distress that these kids experience?’ (R029). Another found data collection difficult ‘when it wasn’t something the family was enjoying’, adding: ‘I think the bloods were frequently bad’ (R040).

##### Confidentiality and data access

Participants expressed some concerns about data confidentiality (‘having watched the X-Files many years ago, one wonders what happens to all your data . . . ’.) but, at the same time, advised ‘not to look into the conspiracy theories too much’ (P035). One mother was worried that government might gain access to biobank data and ‘use it for the wrong purposes’ (P018), citing the example of driving licence restrictions being imposed on people with diabetes. Another mentioned insurance concerns: ‘That they’ll be used . . . to say ‘we’re not insuring this person’’ (P008). Employment was also raised as a potential concern: ‘Having it on a file somewhere with his name to say that he is ASD . . . And that would prevent employment or prevent any workers comp[ensation] stuff if someone went on stress-leave’ (P021). Another mother raised the issue of ownership: ‘I just hope that we don’t lose ownership of our genes’ (P012).

Other parents, however, expressed confidence about data confidentiality saying they had trust: (1) in the government: ‘I feel that the research we do here is vetted’ (P019); (2) in the ethics protocols of universities: ‘I do feel much more confident that they have our rights first and foremost’ (P015); and (3) in the specific ethical safeguards offered by the Biobank: ‘I did read up on that . . . there were very strict guidelines’ (P044).

##### Contesting eugenics, supporting neurodiversity

A major concern participants expressed about contributing to the Biobank was that the research may be used to identify biomarkers for eugenic purposes: ‘I’d hate to see us get to the point where people are able to diagnose in utero that autism is there, and for people to then say ‘well, that pregnancy shouldn’t continue’’ (P027); ‘Would people then start to breed out autism?’ (P039).

Researchers were well aware that autism biobanks have been criticised as having eugenic implications. They also knew that some families were worried: ‘I think the concern for anyone participating in genetic research is: “What will you use my data for? . . . like eugenics, hence eliminate autism?” We were clear this was not what our research would result in’ (R033). Others were more succinct: ‘We don’t want to expunge autism from the gene pool . . . that’s just absolutely nonsense’ (R029). Indeed, the same researcher distanced themselves from any interest in causation: ‘It’s not important to me anymore why kids have developed the way they have’.

Parents who expressed concerns about the potential eugenic implications of biobank research often spoke about the value of their own children and of autistic people more generally. One mother of two autistic children said:

I think that autistic people can bring a lot of good to the world in terms of the way that they can think about things differently to neurotypicals, and potentially come up with a lot of solutions to problems that we have . . . I’d like to think that my children can still offer something to the world. (P007)

Some of the autistic children, as well as their siblings, perceived the purpose of the biobank as focused ‘on how spectrum people think’ (A028) or ‘how their brain works and how they see the world’ (A017). An autistic adolescent told us that a good outcome of the biobank research would be to ‘see how we can use their differences to our advantages, so Steve Jobs, for example, had Asperger’s Syndrome . . . So maybe we could have more people like Steve Jobs’ (A028). The idea of valuing autistic people by preserving their DNA was taken up by an autistic adolescent who compared the Biobank to ‘a little Cryofridge of genetics’ (A043), an ark allowing autistic people to survive.

## Discussion

This qualitative study has presented some of the key motivations and concerns of both participants and researchers contributing to one autism biobank. In so doing, it highlights the powerful role that ethical debates play in shaping the participation of researchers, parents and autistic young people in biobank research, including: (1) the potential for biobank research to help correct damaging misconceptions about autism; (2) a more general sense of the importance of scientific endeavour and a need to support it, countered by a series of concerns about how scientific research is sometimes conducted; and (3) a deeply diverse set of expectations and anxieties about the place of genetics in autism research and the implications of genetic research for autistic people’s lives.

Our parents frequently said they wanted an answer to the question of why their child is autistic because they believed that biobank research might help remove some widespread misconceptions. Consistent with Miller et al. (2010), this finding suggests an ‘anticipatory hope’ connected to the idea that there is an as-yet undiscovered biological essence underpinning autism (Hens, 2021). Our mother interviewees particularly felt that finding a ‘cause for autism’ in this way could help to overturn stigma and mother blame. They were aware of how an emphasis on the genetic causation of autism has sometimes been seen as instrumental in discrediting mother-blame (Bumiller, 2009; Farrugia, 2009; Singh, 2015b).

However, this was not straightforward. Women also reported concerns about the development of autism *in utero* or as a result of birth complications, for which some felt partly responsible. Such concerns are part of ongoing debates (Chaste & Leboyer, 2012). Previous research has described how attributing autism causation to *de novo* genetic mutations may buffer against stigma but may also induce feelings of guilt in parents, leading to stigmatisation and conflicts within families (Hens et al., 2016a) linked to the assignation of genetic blame (Miller et al., 2010). Mothers in our study confirmed this complexity, variously hoping that finding a cause for their child’s autism would alleviate guilt but also describing how the emphasis on genetic contributions could be stigmatising.

The potential for biobank research to help correct harmful misconceptions also fed into more general support for the power of conventional science. Many interviewees conceptualised the Biobank as an open scientific resource that could be used for diverse purposes in the future. In so doing, they frequently emphasised their trust in the role of conventional science. This sense of anticipation (Lappé, 2014), of future promise, pervaded the accounts of family members and researchers (Rapp, 2016).

This commitment to conventional science stood in sharp contrast to the competing claims and controversies about complementary and alternative treatments and therapies (Klein & Kemper, 2016; Lilley, 2011) and childhood vaccination hesitancy fuelled by the fraudulent study by Wakefield et al. (1998) (Godlee et al., 2011). Parents in our study were keen to distance themselves from ‘conspiracy theories’ and align themselves with conventional science. Some of the autistic young people we interviewed expressed aspirations to be scientists. Parents encouraged their children to contribute to the Biobank to foster these interests and aspirations.

Nonetheless, criticisms of conventional scientific practice and the need to make improvements did emerge. Some participants spoke about wanting to be genuinely included in the research process, and treated as a person rather than merely a source of data. Some researchers, too, acknowledged the ethical importance of developing a sense of reciprocity with participants (Tabor et al., 2011). These anxieties revolved around the moral need to acknowledge the value of ‘clinical labour’ (Mitchell & Waldby, 2010) in what is a statistically driven big data enterprise.

Parents and autistic young people also noted various risks, including the possibility of genetic discrimination and concerns about data confidentiality. As genetic testing and biobanks have become commonplace, these concerns have become urgent (Feldman & Darnell, 2013; Kim et al., 2021), prompting calls for greater public engagement in these bioethical issues (Burgess et al., 2008). Both researchers and family members further expressed strong concerns about the distress of blood draws for autistic children, with some parents describing traumatic experiences of extraction as previously outlined by Hens et al. (2016a; see also Asbury et al., 2023). This issue raises important questions about thresholds for minimal risk or harm in autism biobanks (see Tabor et al., 2011).

The sharpest ethical concern about biobank research in broader public debate concerns the fundamental question of whether it might lead to deeply troubling, even ‘eugenicist’, consequences. Some worry that the focus on genetic causes of autism potentially devalues autistic lives, rendering them as ‘ab’ or ‘sub’ normal and requiring pre-birth intervention. As such, Singh (2015a) has described ambivalence and resistance to genetic testing from autistic adults in the United States – a sentiment partly shared by participants in Byres et al. (2023). Certainly, a key concern of autistic advocates expressed in the Spectrum 10K controversy is that biobank data ‘may be used for eugenic purposes in the future such as preventing autistic or otherwise neurodivergent people from existing’ (Chapman et al., 2021).

All these themes emerged in our research. For their part, some Biobank researchers distanced themselves from issues of autism causation, while also describing concerns about eugenics as ‘nonsense’. Researchers emphasised what they perceived as positive outcomes of future research, including the development of pharmacological treatments for co-occurring conditions and earlier identification and intervention. Even so, some envisioned a future in which ‘preventive measures’ based on genetic screening would be offered to families with ‘at-risk’ children. Parents were divided on the desirability of this, as is widely suggested in the existing international literature (Chen et al., 2013, 2015; Johannessen et al., 2017; Xu et al., 2016; Zhang et al., 2022). Akin to Asbury et al. (2023), parental support for genetic testing for autism was most clearly expressed by parents caring for autistic children with co-occurring ID, suggesting that views of autism as predominantly characterised by impairment shape opinions about the desirability of genetic screening. This might reflect broader societal values. It might also relate to the challenges often faced by parents in caring for their autistic children with ID and other co-occurring conditions, especially in the context of limited support services for these individuals and their families (Lord et al., 2021) and the stigma associated with such disability (Mitter et al., 2019).

More strikingly still, some of the autistic young people in our study also voiced support for the prevention or ‘cure’ of autism, especially for ‘the more serious cases’. It is possible that these autistic young people conceptualised autism negatively in response to the stark challenges they or others they know have faced and to the prevailing stigma often linked to being autistic and having co-occurring ID (Mitter et al., 2019). Negative self-conceptions and ambivalence about autistic identity, resulting at least partially from stigmatising attitudes, have been noted in the literature with autistic adults (Bachmann et al., 2019; Botha et al., 2022; Botha & Frost, 2020; Lilley et al., 2022; Schneid & Raz, 2020) and adolescents (den Houting et al., 2021; Humphrey & Lewis, 2008).

Positive views of genetic research were not shared by all, however. Some parent interviewees found the idea of the development of biomarkers leading to reproductive choice problematic, explicitly raising the concern that people might ‘start to breed out autism’. Tabor and colleagues (2011) have previously reported that parents of autistic children may view the use of genetic information for reproductive planning as a potential harm to autistic children and society in general. Our parents also gave voice to these objections (Buchanan, 1996), worrying that the idea of developing prenatal testing and selective termination practices would assign a negative value to their autistic children. These parents highlighted the uniqueness and the value of their children. Some authors (Cascio, 2012; Cost et al., 2021) have documented the increasing influence of the neurodiversity paradigm on parents of autistic children. One autistic young man, a Jurassic Park fan, even flipped concerns about the eugenic implications of biobanks on their head, seeing the whole point as being to preserve his valuable DNA for future generations.

### Limitations

This study has several limitations. Interviewees all contributed to a single autism biobank. Given their decision either to donate biological and phenotypic materials or to work as researchers, their views of autism biobanks are highly likely to be positive, highlighting benefits rather than harms. Furthermore, the study was retrospective, with some participants having contributed to the Biobank several years prior to interview. Given the time lag between donation and interview, recall bias and changing viewpoints need to be considered.

### Conclusion

Despite these limitations, our multi-informant approach offers insights into the diverse motivations, viewpoints and experiences of participants and researchers contributing to one autism biobank. By identifying why various actors contribute to autism biobank research and their experiences of doing so, our analysis points to both anxieties and optimism about the potential of autism science. This reminds us of the importance of attending to diverse views when seeking to understand the decisions to contribute and the expectations of autism biobanks. The future direction of autism science is likely to remain an issue of significant contestation in the years ahead.

## Supplemental Material

sj-docx-1-aut-10.1177_13623613231203938 – Supplemental material for Contributing to an autism biobank: Diverse perspectives from autistic participants, family members and researchers Supplemental material, sj-docx-1-aut-10.1177_13623613231203938 for Contributing to an autism biobank: Diverse perspectives from autistic participants, family members and researchers by Rozanna Lilley, Hannah Rapaport, Rebecca Poulsen, Michael Yudell and Elizabeth Pellicano in Autism
