# Supplementary material for: Contributing to an autism biobank: Diverse perspectives from autistic participants, family members and researchers
Source: Autism. 2023 Oct 26;28(7):1719–31. doi: 10.1177/13623613231203938 (PMC11191664; doi:10.1177/13623613231203938)
Supplement: sj-docx-1-aut-10.1177_13623613231203938 – Supplemental material for Contributing to an autism biobank: Diverse perspectives from autistic participants, family members and researchers [file sj-docx-1-aut-10.1177_13623613231203938.docx]

**Supplementary Table 1.** Participant interview schedules: primary questions

| **Parents** | **Autistic children and adults** | **Siblings** | **Researchers** |
| --- | --- | --- | --- |
| *About your child*   - Tell me a bit about your child. - Can you tell me about your experience of getting a diagnosis for your child? - Which therapies have you and your child taken part in over the years? - Thinking about your child’s future, do you have a plan or ideas about what he/she might do when they finish school? - Do you have any views about what causes autism and how would you describe autism to someone who didn’t know much about it?   *About your participation in the Biobank*   - What do you think the Australian Autism Biobank is trying to achieve? - Can you tell me a bit about your understanding of genetics? - It would be great if you could take us through what you and your family were asked to do for the biobank – from the moment you said yes to when you had completed the sessions? - What has your experience been like with the researchers involved and have you received any feedback about your participation? - Have you done, or are you thinking about doing, any other genetic testing? - Have you or your family been involved with other autism research? - What are your views about autism research in general? | - Can you tell me a little about yourself? - Can you tell me a bit about your family? - You were asked to participate in a study recently because you have a diagnosis of autism. Can you tell me a bit about what you think autism is? - Why do you think some people are autistic and some people are not autistic? - You took part in something called the Australian Autism Biobank. Do you know what that research project is looking at? - Who asked you if you were willing to take part in that research? - What were you asked to do in the Australian Autism Biobank study? - What can you tell me about genetics? - Would you like to find out about the results of the Biobank study? - What are you hoping to do in the future? | - Can you tell me a little about yourself? - Can you tell me a bit about your family? - Your brother/sister is diagnosed with autism. Can you tell me what autism is? - What do you think causes autism? - You took part in something called the Australian Autism Biobank. Do you know what that research project is looking at? - Who asked you if you were willing to take part in that research? - What were you asked to do in the Australian Autism Biobank study? - What can you tell me about genetics? - Would you like to find out about the results of the Biobank study? - What are you hoping to do in the future? | *About your job*   - Tell me a bit about your current job. - How would you describe autism? - How did you first become involved in autism research?   *About your role in the Australian Autism Biobank*   - What is your role with the Australian Autism Biobank? - Are you involved with other types of genetic and/or autism research? - How will the Australian Autism Biobank contribute to understandings of autism? - What do you think are the main ethical issues to consider with this kind of research? - What kinds of autism research would you like to see prioritised? |
